# Can a pill (Ca^2+^ channel blockade) rescue the vascular endothelium from ageing?

**DOI:** 10.1113/EP091824

**Published:** 2024-03-18

**Authors:** Seth W. Holwerda

**Affiliations:** ^1^ Department of Anesthesiology, Department of Cell Biology and Physiology, KU Diabetes Institute University of Kansas Medical Center, KC Kansas City USA

**Keywords:** ageing, calcium channel, endothelium, FMD, hypertension

1

Function of the vascular endothelium is impaired with ageing, which poses a challenge for a healthy and active lifestyle by restricting limb blood flow and increasing risk of cardiovascular disease. Leg blood flow is reduced by approximately 26% in older men compared with young men (Dinenno et al., 1999), but with habitual endurance exercise, leg blood flow can be preserved. In the current issue of *Experimental Physiology*, Iepsen and colleagues took a pharmacological approach and examined the extent to which low voltage T‐type Ca^2+^ channel blockade improves function of the vascular endothelium in ageing men (Iepsen et al., 2024). Low voltage T‐type Ca^2+^ channels play a significant role in vascular resistance and have increasingly become a therapeutic target for hypertension. However, controversy exists regarding the exact role that T‐type Ca^2+^ channels play in the function of the vascular endothelium in humans.

Studies in humans targeting the low voltage T‐type Ca^2+^ channel for improving function of the endothelium have been scarce. Results from a randomized, double‐blinded clinical trial (Oshima et al., 2005) showed significant improvement in endothelium‐dependent dilatation in patients with essential hypertension following 12 weeks of T‐ and L‐type Ca^2+^ channel blockade (efonidipine), but not after 12 weeks of L‐type Ca^2+^ channel blockade alone (nifedipine). Thus, by deduction, the study paradigm isolates the influence of T‐type Ca^2+^ channel blockade. The work by Iepsen and colleagues includes the same study approach but instead focuses on older men rather than patients with hypertension. Iepsen et al. report that endothelium‐dependent dilatation was significantly increased in older men following 8 weeks of efonidipine, but not nifedipine. Endothelium‐dependent dilatation has significant clinical value because it is inversely related to future risk of cardiovascular disease.

Two principal elements of the study by Iepsen et al. should be mentioned. First, the study design includes randomization and double blinding, which remains the gold standard approach for demonstrating efficacy. A placebo condition was not included; however, nifedipine served as a necessary and sufficient comparator for efonidipine. Second, study participants did not have hypertension, nor did they demonstrate a statistically significant reduction in blood pressure (BP) following T‐type Ca^2+^ channel blockade. This is significant because it suggests that T‐type Ca^2+^ channel blockade does not require a major reduction in BP to elicit an improvement in endothelium‐dependent dilatation. Endothelium‐dependent dilatation declines after age 40, while systolic BP tends to increase thereafter. Hence, T‐type Ca^2+^ channel blockade may have potential as a prophylactic for age‐related reductions in function of the endothelium even before BP begins to increase if sufficient evidence bears out in future clinical studies. In ageing adults, it is critical to mitigate hypertension and its contributing factors (e.g., impaired endothelium‐dependent dilatation) because hypertension is independently associated with damage to end organs, such as the brain, and contributes to risk of stroke and dementia.

T‐type Ca^2+^ channels are thought to make a larger contribution to vascular resistance in the smaller peripheral arteries compared with large peripheral arteries. Indeed, the relative expression of T‐ and L‐type Ca^2+^ channels is inverse, where T‐type Ca^2+^ channels are expressed more in the small arteries and L‐type Ca^2+^ channels are expressed more in large arteries. T‐type Ca^2+^ channels are also regulated by numerous G‐protein‐coupled receptors, which means that changes in the milieu (e.g., angiotensin II) may potentially alter T‐type Ca^2+^ channel control of vascular function. A major obstacle to clinical research is the variation in the α_1_ subunit of the T‐type Ca^2+^ channel (Cav.3.1, Cav.3.2 and Cav.3.3) because it creates difficulty in targeting the drug. However, development of knockout mice has made it possible to determine the functional role of T‐type Ca^2+^ channel subtypes. Evidence suggests that Cav3.2 channels may affect vasodilatation through a vascular smooth muscle cell‐related mechanism (Chen et al., 2003), whereas Cav.3.1 may affect vasodilatation through a nitric oxide mechanism (Svenningsen et al., 2014). Iepsen and colleagues provide evidence in humans that T‐type Ca^2+^channel blockade improves function of the endothelium through a nitric oxide mechanism rather than a vascular smooth muscle cell‐related mechanism because endothelium‐dependent dilatation was significantly increased while dilatation in response to a nitric oxide donor was unchanged.

While applauding the authors for performing a highly challenging study given the invasive nature of the assessments, we should also note the limitations and study outcomes that do not relieve controversy regarding the role of T‐type Ca^2+^ channels in the function of the endothelium. First, despite higher endothelium‐dependent dilatation following treatment, a parallel increase in plasma nitrite and nitrate (NOx) concentration was not observed. Nitric oxide production occurs primarily on the arterial side of the circulation, and increased concentration of NOx from arterial blood reflects higher endothelium‐dependent dilatation. Thus, an increase in NOx was expected. Second, a comparison of biopsy from the vastus lateralus before and after treatment did not provide support for upregulation of endothelial nitric oxide synthase (eNOS). An alternative viewpoint is that eNOS is phosphorylated instead of upregulated following T‐type Ca^2+^ channel blockade, indicating greater functional activity of eNOS rather than increased eNOS content. However, phosphorylated eNOS was not assessed by Iepsen et al., making this a notable limitation of the study.

Advancements in the field often create more questions than answers, as does the study by Iepsen et al. For instance, it is unclear if an age threshold exists where T‐type Ca^2+^ channel blockade does not improve function of the endothelium. Also, additional studies will be required to determine the extent to which T‐type Ca^2+^ channel blockade improves age‐related declines in function of the endothelium in women. No apparent sex differences were noted in a previous study that included adults with essential hypertension (Oshima et al., 2005), suggesting that T‐type Ca^2+^ channel blockade could potentially be beneficial in ageing women.

In summary, Iepsen et al. provide supporting evidence in humans that T‐type Ca^2+^ channels contribute independently to function of the endothelium. It is quite notable that a +33% increase in endothelium‐dependent dilatation was observed in the absence of a structured physical exercise regimen, which has been shown to increase endothelium‐dependent dilatation in middle aged and older men by 30% (DeSouza et al., 2000). These data provide reason to be optimistic about larger clinical trials focused on nitric oxide bioavailability in the vascular endothelium following T‐type Ca^2+^ channel blockade in middle‐aged and older adults.

## AUTHOR CONTRIBUTIONS

Sole author.

## CONFLICT OF INTEREST

No conflicts of interest, financial or otherwise, are declared by the author.
